# *PTPN22 *polymorphism and anti-cyclic citrullinated peptide antibodies in combination strongly predicts future onset of rheumatoid arthritis and has a specificity of 100% for the disease

**DOI:** 10.1186/ar1868

**Published:** 2005-12-22

**Authors:** Martin Johansson, Lisbeth Ärlestig, Göran Hallmans, Solbritt Rantapää-Dahlqvist

**Affiliations:** 1 Department of Rheumatology, University Hospital, Umeå, Sweden; 2 Department of Nutritional Research, University Hospital, Umeå, Sweden

## Abstract

We analysed relationships between the *PTPN22 *1858 polymorphism and antibodies to cyclic citrullinated peptide (CCP), rheumatoid factors (RFs) and the shared epitope (SE) gene (HLA-DRB1*0404 or 0401) and determined their combined predictive value for rheumatoid arthritis (RA) in individuals who subsequently developed RA. This case-control study was nested within the Medical Biobank of Northern Sweden. Patients with RA (n = 92) were identified from amongst blood donors antedating onset of disease by a median of 2.4 (interquartile range 1.2 to 4.9) years. Matched controls were selected randomly from the same cohorts (n = 368). Anti-CCP antibodies and RFs were determined using enzyme-linked immunoassays. Genotyping was performed using an ABI PRISM 7900HT instrument and HLA-SE genes were identified using PCR sequence-specific primers. The 1858T allele and also carriage of T were associated with future onset of RA (odds ratio (OR) = 2.29, 95% confidence interval (CI) 1.45–3.61 and OR = 2.64, 95% CI 1.56–4.47, respectively). The combination of the 1858T variant and anti-CCP antibodies gave 100% specificity for the disease. None of the 368 controls expressed this combination. The *PTPN22 *1858T variant and anti-CCP antibodies were clearly associated (OR = 3.80, 95% CI 1.51–9.57). A combination of the *PTPN22 *1858T variant and anti-CCP antibodies gave a much higher relative risk (>132.03) for developing RA than the combination of the T variant and HLA-SE (OR = 7.85). The *PTPN22 *1858T variant was associated with future development of RA. There was an association between the T variant and anti-CCP antibodies and their combination, found only among pre-patients, gives a very high relative risk for development of RA. The combination gave a specificity of 100% for diagnosing RA.

## Introduction

A single nucleotide polymorphism in the *PTPN22 *gene encoding the lymphoid protein tyrosine phosphatase (Lyp) has recently been found to be associated with several autoimmune disorders. The *PTPN22 *1858C/T polymorphism was originally associated with type I diabetes [1] and later with other autoimmune diseases, for example, systemic lupus erythematosus [2], Graves' disease [3] and Hashimoto thyroiditis [4]. Several studies report an association of it with rheumatoid arthritis (RA) [4-10]; the association was primarily with sero-positive disease [5,8] but two recent studies show association with both sero-positive and sero-negative RA [9,10]. This association with RA appears to be the most robust and reproducible genetic association outside the human leukocyte antigen (HLA) region. In several of the autoimmune diseases associated with the *PTPN22 *polymorphism, the appearance of autoantibodies precedes the development of overt clinical disease by months or years [11-13]

We previously reported that antibodies against cyclic citrullinated peptide (CCP) and IgA-rheumatoid factor (RF) predict development of RA by a median of 2.5 years [14]. The presence of anti-CCP antibodies together with HLA shared epitope (HLA-SE) genes (HLA-DRB1*0404/0401) increased the relative risk for development of RA [15].

In this study, we analysed the *PTPN22 *1858C/T polymorphism in relation to anti-CCP antibodies, RFs (IgM, IgG and IgA) and HLA-SE gene carriage in individuals who had donated blood before development of RA. The predictive effects of the genes and antibodies were then evaluated.

## Materials and methods

A nested case-control study was performed within the Medical Biobank of Northern Sweden of the Northern Sweden Health and Disease Study (NSHDS). All adults in Västerbotten county were invited to participate; consequently, the cohort is population-based and no individual was excluded. The Medical Biobank, conditions for recruitment into the cohorts, and the collection and storage of blood samples have previously been described in detail [14]. To identify whether our patients diagnosed with RA (according to the American College of Rheumatology 1987 criteria [16]) at the early arthritis clinic within the Department of Rheumatology, University Hospital, Umeå, had donated blood samples before any symptoms of joint disease, the register of RA patients was co-analysed on two occasions with those of the NSHDS Medical Biobank. Ninety-two individuals (69 women and 23 men) were identified; in each case the date of onset of symptoms of joint disease was available. For every case (for instance, pre-patient), four controls were randomly selected from within the NSHDS registers and matched for sex, age at the time of blood sampling and rural or urban residence. A total of 368 controls (276 women and 92 men) were selected. The mean age of the pre-patients and of the controls at the time of blood sampling was the same, namely 53 years (range 30 to 69 years). The median sampling time before onset of symptoms of joint disease was 2.4 years (interquartile range 1.2 to 4.9 years). On average, the diagnosis of RA was established 7.8 months (interquartile range 5 to 10 months) after the first symptoms of joint disease. The mean age at the onset of disease was 56.0 years (range 37 to 68 years). At the time of the study, the median disease duration since diagnosis was 3.0 years (interquartile range 1.9–5.5 years). The Ethics Committee at the University Hospital, Umeå, approved this study and all participants gave their written informed consent.

HLA-DRB1 genotyping was performed using PCR sequence-specific primers from the DR low-resolution and DRB1*04 subtyping kits (Olerup SSP AB, Saltsjöbaden, Sweden) according to the previously described method [15]. The HLA-SE genes were defined as DRB1*0404 and DRB1*0401. Two controls were randomly selected for each pre-patient. The HLA-DRB1 typing was successful for 90 pre-patients and 173 controls. Anti-CCP antibodies (n = 89 for pre-patients and n = 353 for controls) and the RFs of IgM, IgG and IgA isotypes (in the first 59 pre-patients identified and 236 controls) were determined using enzyme-linked immunoassays as previously described [14].

DNA was extracted from EDTA-treated whole blood using a standard method. The *PTPN22 *1858C/T polymorphisms (single nucleotide polymorphism number is rs2476601) were determined with the 5' nuclease assay. DNA was unavailable for *PTPN22 *genotyping for three patients and two controls. Primers and probes were designed by Applied Biosystems (Foster City, CA, USA). The forward primer was 5'-CAACTGCTCCAAGGATAGATGATGA-3'and the reverse primer was5'-CCAGCTTCCTCAACCACAATAAATG-3'. The probes were labelled at their 5' ends with FAM™ (the C allele) and VIC™ (the T allele) and the 3' ends contained quenchers and minor groove binders. The probe for the A allele was 5'-6FAM-TCAGGTGTCCGTACAGG-MGB-Q-3' and for the G allele 5'-VIC-TCAGGTGTCCATACAGG-MGB-Q-3'. Primers and probes were mixed with TaqMan^® ^Universal PCR Master Mix, No AmpErase^® ^UNG (Applied Biosystems, Foster City, CA, USA) and added to 96-well microtitre plates, each well of which contained 10 ng of air-dried DNA. The PCR-reactions were performed according to the manufacturer's instructions and detection of the different genotypes made using an ABI PRISM^® ^7900HT Sequence Detector System (Applied Biosystems). Data were processed using SDS 2.1 software (Applied Biosystems). The different genotypes were verified by comparison with controls of known genotype. Genotyping was unsuccessful for six controls.

The Chi-square test was used for testing categorical data between groups. To assess the utility of the various antibodies and genes in detecting prospective RA patients, sensitivity, specificity and likelihood values were calculated. Conditional logistic regression models were used to estimate the predictive values of the antibodies analysed, the *PTPN22 *1858T variant, the RFs, and HLA-SE gene carriage for RA. Odds ratios (ORs) were calculated with 95% confidence intervals (CIs). All p-values refer to a two-sided test and a *p* value ≤0.05 was considered statistically significant. The calculations were performed using the SPSS package (SPSS for Windows 12.0: SPSS Inc., Chicago, IL, USA). Power calculations based on the frequency among our controls and among RA patients from other studies showed that four controls per patient would be sufficient.

## Results

The genotype and allele distribution of the *PTPN22 *1858C/T single nucleotide polymorphism were in agreement with the Hardy-Weinberg equilibrium among both the pre-patient group and the control population. The genotype frequencies differed significantly between the pre-patients and the controls (χ^2 ^= 15.37, 2 degrees of freedom, p = 0.0005) (Table 1).

Carriage of the *PTPN22 *1858T variant (CT+TT) was significantly increased in pre-patients compared with controls, as was the frequency of the T allele (OR = 2.64, 95% CI 1.56–4.47 and OR = 2.29, 95% CI 1.45–3.61, respectively) (Table 1). The CC genotype was significantly decreased (OR = 0.38, 95% CI 0.22–0.64). Carriage of T was associated with the development of RA (χ^2 ^= 15.21, *P* = 0.0001) as was carriage of the HLA-SE gene (χ^2 ^= 7.28, *P* = 0.007).

The sensitivity of *PTPN22 *1858T carriage for identifying pre-patients was lower compared with HLA-SE but of the same magnitude as presence of anti-CCP antibodies (Table 2). The specificity, however, was greater with *PTPN22 *1858T carriage than with HLA-SE but less than with anti-CCP antibodies. The combination of anti-CCP antibodies and *PTPN22 *T carriage gave a specificity of 100%. The highest likelihood ratio was for anti-CCP antibodies (26.2), followed by the different RFs (3.7 to 8.3). The *PTPN22 *1858T variant had a higher likelihood ratio than HLA-SE (2.0 and 1.5, respectively). The combination of carrying both gene variants, however, gave a higher likelihood ratio (3.4).

Carriage of the *PTPN22 *1858T variant and presence of anti-CCP antibodies were significantly associated in the pre-patients (χ^2 ^= 8.39, *P* = 0.004, OR = 3.80, 95% CI 1.51–9.57). None of the controls carrying the T variant had anti-CCP antibodies. Nor was there any significant relationship between carriage of the *PTPN22 *1858T variant and any of the RF isotypes or HLA-SE, either in pre-patients or in controls (data not presented). There was no significant relationship between HLA-SE and anti-CCP antibodies in either of the groups (data not presented). In multiple conditional logistic regression analysis with carriage of the *PTPN22 *1858T variant and HLA-SE as independent variables, both predicted RA but with the *PTPN22 *1858T variant giving the highest value (OR = 3.51, 95% CI 1.85–6.68 and OR = 2.19, 95% CI 1.22–3.94, respectively).

To analyse the relative risk of developing RA, carriage of the *PTPN22 *1858T variant was combined with anti-CCP antibodies, RFs (IgG-RF, IgA-RF and IgM-RF) and with HLA-SE in conditional logistic regression analyses. The combination of anti-CCP antibodies and carriage of the *PTPN22 *1858T variant gave a very high OR compared with not having any of them. As none of the controls had this combination, the OR was calculated assuming one individual did, resulting in a value of 132.03, although the relative risk was actually infinite (Table 3). Carriage of the T variant combined with RFs gave the highest OR for IgA-RF (OR = 21.42) followed by IgM-RF (OR = 10.70) compared with individuals not having any of them. The combination of the T variant with IgG-RF did not give a significant relative risk. The combination of the *PTPN22 *1858T variant with HLA-SE gave an OR of 7.85, which was greater than that for either of them separately (OR = 3.35 and OR = 2.12, respectively), all compared with not having either of them.

## Discussion

This study involved individuals who had donated blood samples to the Medical Biobank of the NSHDS prior to developing any RA symptoms. In these pre-patients who developed RA, there was an association of it with the *PTPN22 *1858C/T polymorphism, consistent with previous reports on RA [4-10]. We also found that the presence of anti-CCP antibodies was significantly associated with carriage of the T variant and there was a greatly increased relative risk for the development of RA in individuals with a combination of the *PTPN22 *1858T variant and anti-CCP antibodies. This relative risk was much higher than with the combination of HLA-SE and anti-CCP antibodies, as we have previously reported [15]. In our previous study, the OR was 66.8 whereas that for the combination of the *PTPN22 *1858T variant and anti-CCP antibodies was >132.03 based on a calculation using one hypothetical control subject as being positive for both *PTPN22 *1858T and anti-CCP antibodies. None of the control subjects with the *PTPN22 *1858T variant were seropositive for anti-CCP antibodies. Anti-CCP antibodies were only present in controls with the 1858CC genotype (n = 5). This could suggest that the *PTPN22 *T variant influences the progression of overt autoimmune disease once autoantibodies, such as anti-CCP antibodies, have developed. This is the first study to show an association between the *PTPN22 *1858T variant and a disease related autoantibody, and that they co-operate to increase the relative risk of developing an autoimmune disease, in this case RA.

Our data suggest that carriage of the *PTPN22 *1858T variant is of greater importance than HLA-SE for the development of RA. There was an increased relative risk of developing RA with the combination of the *PTPN22 *T variant and HLA-SE; however, this relative risk was lower than with the combinations of the T variant with IgA-RF or IgM-RF. With respect to RFs, we have reported higher relative risks for developing RA with the combination of HLA-SE and RFs in a smaller cohort of the same study population [15]. We did not find the significant association with carriage of the T variant and RFs suggested by some studies [5,8] but our results are in concordance with others [9,10].

The function of Lyp, the protein the *PTPN22 *gene encodes, is suggested to be negative regulation of T-cell signalling, as demonstrated in an animal model [17] and in human cell lines [5]. The functional effect of the *PTPN22 *1858 polymorphism on T-cells in humans is yet to be demonstrated. The Lyp protein is expressed in other cell types: B-cells, monocytes, neutrophils, dendritic cells and natural killer cells [5]. In a mouse knockout model lacking the murine homologue of human PTPN22 (PEST domain-enriched tyrosine phosphatase (PEP)), the threshold for T-cell receptor signalling was lowered and the number of effector and memory T-cells increased [17]. The knockout mice also showed an increased number of germinal centres and increased immunoglobulin levels, although autoantibodies were not detected in these animals. Changes in B-cell function were not found, suggesting that the abnormalities reflect a role of T-cell regulation on B-cell differentiation. *PTPN22 *1858T changes codon 620 from arginine into tryptophan. This amino acid change disrupts the binding of Lyp to an intracellular kinase, Csk, which can then no longer inactivate another kinase, Lck, that is involved in T-cell signalling. The result of this missense mutation is a possible loss of negative regulation of T-cell signalling [1,5].

A limitation of this study is the relatively small number of pre-patients identified who later developed RA, which was further reduced in terms of analysis of RFs, and of controls genotyped for HLA-SE. The study cohort (NSHDS) is population-based and no exclusion criteria were applied. The controls could have included two to four individuals with RA (calculated using a prevalence for RA of 0.5% to 1.0%), which could explain the number of individuals among the controls positive for anti-CCP antibodies.

## Conclusion

This study shows a strong association between the *PTPN22 *1858T variant and future development of RA. This association is stronger than that for HLA-SE and is the better predictor for RA. We also show an association between the T variant and anti-CCP antibodies but not RFs. The combination of the *PTPN22 *1858T variant and anti-CCP antibodies was found only among pre-patients, making it a strong predictor for the development of RA, possibly by influencing the progression of an overt autoimmune disease.

## Abbreviations

CCP = cyclic citrullinated peptide; CI = confidence interval; HLA-SE = human leukocyte antigen shared epitope; Lyp = lymphoid protein tyrosine phosphatase; NSHDS = Northern Sweden Health and Disease Study; OR = odds ratio; RA = rheumatoid arthritis; RF = rheumatoid factor.

## Competing interests

The authors declare that they have no competing interests.

## Authors' contributions

MJ was a main investigator and carried out the genotyping, the statistics and contributed to preparation of the manuscript. LÄ participated in the collection of the material and registration of the pre-patient data. GH is responsible for the Medical Biobank of NSHDS and delivered the DNA samples. SR-D is the principal investigator, is responsible for the Biobank samples, designed the investigation, and participated in data collection, statistical analysis and drafting of the manuscript.

## Figures and Tables

**Table 1:** Frequency distribution of the *PTPN22 *1858C/T polymorphism

	Pre-patients (n = 89)		Controls (n = 360)		χ^2^	*P* value	OR	95% CI
	N	%	n	%				

CC	54	60.7	289	80.3	15.21	0.00010	0.38	0.22–0.64
CT	33	37.1	68	18.9	13.54	0.00025	2.52	1.48–4.30
TT^a^	2	2.2	3	0.8	1.30	0.2586	2.74	0.31–20.46
CT + TT	35	39.3	71	19.7	15.21	0.00010	2.64	1.56–4.47
T allele	37	20.8	74	10.3	14.55	0.00013	2.29	1.45–3.61

Frequencies of *PTPN22 *1858C/T genotypes and of the T allele were determined in individuals who later developed rheumatoid arthritis (pre-patients) and in controls.

^a^Fisher's exact test (two-sided). CI, confidence interval; OR, odds ratio.

**Table 2:** Sensitivity, specificity, 95% confidence intervals and likelihood ratios

	Sensitivity		Specificity		Likelihood ratio
	%	95% CI	%	95% CI	

*PTPN22 *CT+TT	39.3	29.6–49.7	80.3	75.9–84.1	2.0
Anti-CCP Abs	37.1	27.5–47.5	98.6	96.9–99.5	26.2
IgG-RF	16.9	8.9–28.1	95.8	92.6–97.8	4.0
IgA-RF	42.4	30.3–55.2	94.9	91.5–97.2	8.3
IgM-RF	22.0	12.8–33.9	94.1	90.5–96.6	3.7
SE (B1*0404 or 0401)	55.6	45.2–65.6	61.8	54.4–68.9	1.5
SE + *PTPN22 *CT+TT	24.1	16.0–34.0	92.9	88.3–96.1	3.4
Anti-CCP Abs + *PTPN22 *CT+TT	22.1	14.3–31.8	100.0	-	-

Sensitivity, specificity, 95% confidence intervals (CIs) and likelihood ratios were determined for the presence of the *PTPN22 *1858T variant (CT+TT), anti-cyclic citrullinated peptide antibodies (anti-CCP Abs), rheumatoid factors IgM-RF, IgG-RF and IgA-RF, and carriage of human leukocyte antigen shared epitope (HLA-SE) in pre-patients and matched controls.

**Table 3:** Conditional logistic regression analyses of combinations of genes and antibodies

Combination of variables	Pre-patients		Controls		OR	95% CI
	n	%	n	%		

*PTPN22 *CT+TT^- ^+ anti-CCP Abs^-^	39	45.3	272	78.4	1.00	
*PTPN22 *CT+TT^+ ^+ anti-CCP Abs^-^	15	17.5	70	20.2	1.20	0.62–2.35
*PTPN22 *CT+TT^- ^+ anti-CCP Abs^+^	13	15.1	5	1.4	16.61	4.68–58.97
*PTPN22 *CT+TT^+ ^+ anti-CCP Abs^+^	19	22.1	0^a^	0.0	132.03^a^	17.84–2720.91^a^
						
*PTPN22 *CT+TT^- ^+ IgG-RF^-^	27	48.2	175	76.1	1.00	
*PTPN22 *CT+TT^+ ^+ IgG-RF^-^	19	33.9	46	20.0	2.47	1.26–4.85
*PTPN22 *CT+TT^- ^+ IgG-RF^+^	9	16.1	6	2.6	10.08	3.00–33.94
*PTPN22 *CT+TT^+ ^+ IgG-RF^+^	1	1.8	3	1.3	1.50	0.15–14.84
						
*PTPN22 *CT+TT^- ^+ IgA-RF^-^	23	41.1	171	74.3	1.00	
*PTPN22 *CT+TT^+ ^+ IgA-RF^-^	11	19.6	47	20.4	1.55	0.69–3.49
*PTPN22 *CT+TT^- ^+ IgA-RF^+^	13	23.2	10	4.4	9.23	3.31–25.76
PTPN22 CT+TT^+ ^+ IgA-RF^+^	9	16.1	2	0.9	21.42	4.45–103.16
						
*PTPN22 *CT+TT^- ^+ IgM-RF^-^	28	50.0	170	73.9	1.00	
*PTPN22 *CT+TT^+ ^+ IgM-RF^-^	16	28.6	46	20.0	1.97	0.98–4.04
*PTPN22 *CT+TT^- ^+ IgM-RF^+^	8	14.3	11	4.8	4.65	1.65–13.13
*PTPN22 *CT+TT^+ ^+ IgM-RF^+^	4	7.1	3	1.3	10.70	1.78–64.23
						
*PTPN22 *CT+TT^- ^+ SE^-^	24	27.6	86	50.6	1.00	
*PTPN22 *CT+TT^+ ^+ SE^-^	14	16.1	18	10.6	3.35	1.34–8.26
*PTPN22 *CT+TT^- ^+ SE^+^	28	32.2	54	31.8	2.12	1.06–4.25
*PTPN22 *CT+TT^+ ^+ SE^+^	21	24.1	12	7.0	7.85	3.03–20.30

Results of conditional logistic regression analyses of carriage of the *PTPN22 *1858T variant (CT + TT), HLA shared epitope (SE), anti-cyclic citrullinated peptide antibodies (anti-CCP Abs) or rheumatoid factors (RFs) of IgG, IgM, or IgA isotype for the prediction of rheumatoid arthritis in individuals who later developed the disease and matched controls. ^a^Calculations made with a hypothetical control individual positive for both the *PTPN22 *1858T variant and anti-CCP antibodies.
